# EV‐D68 infection in children with asthma exacerbation and pneumonia in Mexico City during 2014 autumn

**DOI:** 10.1111/irv.12384

**Published:** 2016-03-31

**Authors:** Joel A. Vazquez‐Perez, Jose E. Ramirez‐Gonzalez, Yazmin Moreno‐Valencia, Victor A. Hernandez‐Hernandez, Jose A. I. Romero‐Espinoza, Manuel Castillejos‐Lopez, Andres Hernandez, Rogelio Perez‐Padilla, Lizbeth E. Oropeza‐Lopez, Noe Escobar‐Escamilla, Maribel Gonzalez‐Villa, Alejandro Alejandre‐Garcia, Justino Regalado‐Pineda, Patricio Santillan‐Doherty, Irma Lopez‐Martínez, Alberto Diaz‐Quiñonez, Jorge Salas‐Hernandez

**Affiliations:** ^1^ Instituto Nacional de Enfermedades Respiratorias Ismael Cosio VillegasMexico CityMexico; ^2^ Instituto de Diagnostico y Referencia EpidemiologicosManuel Martinez BaezMexico CityMexico; ^3^ Facultad de MedicinaUNAMMexico CityMexico

**Keywords:** Enterovirus D68, epidemiology, outbreak, pediatric, respiratory viruses

## Abstract

**Background:**

Human enterovirus D68 (EV‐D68) recently caused an increase in mild‐to‐severe pediatric respiratory cases in North America and some European countries. Even though few of these children presented with acute paralytic disease, direct causal relationship cannot yet be assumed.

**Objectives:**

The purposes of this report were to describe the clinical findings of an outbreak of EV‐D68 infection in Mexico City and identify the genetic relationship with previously reported strains.

**Patients/Methods:**

Between September and December 2014, 126 nasopharyngeal samples (NPS) of hospitalized children <15 years of age with ARI were tested for the presence of respiratory viruses using a multiplex RT‐qPCR and EV‐D68‐specific RT‐qPCR. Clinical, epidemiological, and demographic data were collected and associated with symptomatology and viral infections. Phylogenetic analyses were performed using VP1 region.

**Results:**

Enterovirus/rhinovirus infection was detected in 40 patients (31·7%), of which 24 patients were EV‐D68‐positive. EV‐D68 infection prevailed over September and October 2014 and was associated with neutrophilia and lymphopenia, and patients were more likely to develop hypoxemia. Phylogenetic analyses showed that Mexican EV‐D68 belongs to the new B1 clade.

**Conclusions:**

This is the first EV‐D68 outbreak described in Mexico and occurred few weeks after the United States reported similar infections. Although EV‐D68 belongs to new B1 clade, no neurological affection was observed.

## Background

*Picornaviridae* family is an extensive group of viruses causing enteric, neurological, and respiratory diseases. *M*embers of this family including rhinoviruses A, B and C, coxsackievirus, echovirus, and enterovirus are responsible for important pediatric enteric and respiratory diseases. Several reports have shown that rhinovirus C is associated with asthma exacerbations.^1^, ^2^, ^3^, ^4^ Non‐polio enteroviruses such as coxsackievirus A16 and enterovirus 71 have been associated with hand, foot and mouth disease in the United States and Asia,^5^ whereas coxsackievirus A24 and enterovirus 70 have been related to conjunctivitis outbreaks and echoviruses 13, 18, and 30 have caused outbreaks of viral meningitis in the United States.^6^

In the late summer of 2014, clusters of severe respiratory infections due to enterovirus D68 had been reported in children throughout the United States, Canada, and the Netherlands.^7^ These children presented with asthma exacerbation and were generally characterized by low‐grade or absent fever, wheezing, dyspnea, and hypoxia.^8^, ^9^ Severe cases of the disease needed mechanical ventilation, and on rare occasions, children developed acute focal limb weakness with non‐enhancing spinal cord lesions following the respiratory illness.^8^, ^9^

The purposes of this report were to describe the clinical findings of an EV‐D68 infection outbreak in Mexico City and identify genetic relationship with the strains reported in United States, Europe, and Asia. During September, we detected an increase in enterovirus/rhinovirus identification on respiratory samples from hospitalized children with pneumonia or asthma exacerbation (none with paralysis), of which 24 were EV‐D68‐positive.

## Patients and methods

### Study population and clinical data

All samples used in the study were collected from children under 15 years of age who presented with clinical signs of acute respiratory infection (ARI) at the Pediatrics Unit of the National Institute of Respiratory Diseases (INER) in Mexico City, a referral center for respiratory diseases mainly attending patients with no health insurance coverage. A total of 126 nasopharyngeal swabs (NPS) were collected from hospitalized patients with ARI, all with difficult breathing or hypoxemia (oxygen saturation < 90% on room air), between September 2014 and December 2014. Demographic data and clinical symptoms of the enrolled patients were obtained from clinical charts including clinical diagnosis, age, gender, signs and symptoms at admission, underlying medical conditions, antibiotic usage prior to admission, physical examination, laboratory findings, length of hospital stay, oxygen requirement, and results from bacterial molecular detection.

The patients were separated into two groups: a) those with pneumonia (all ARI including opacities in the chest roentgenogram); and b) those with wheezing and/or asthma but no chest roentgenogram opacities.

For all children, their legal guardians provided written informed consent for sample donation and allowed diagnostic tests to be performed. All participants assented to participate in the study.

### Viral nucleic acid extraction and EV‐D68 detection

Total nucleic acids were extracted from samples using a high‐throughput automated extraction system (MagNa Pure; Roche, Indianapolis, USA). Multiplex reverse transcription‐polymerase chain reaction (RT‐qPCR) was standardized to detect the main respiratory viruses (influenza, parainfluenza, bocavirus, adenovirus, metapneumovirus, coronavirus, respiratory syncytial virus, and enterovirus/rhinovirus), by high‐throughput gene expression analysis using 48·48 Dynamic Array integrated fluidic chips on the BioMark platform (San Francisco, CA, USA). Subsequent identification of enteroviruses/rhinovirus was performed by semi‐nested RT‐PCR, to obtain a 154‐bp fragment using primers EV1/EV3 in the first round and EV2/EV3 in the semi‐nested reaction as previously described.^10^ To identify EV‐D68 by RT‐qPCR method, a specific forward primer, compatible with EV3 primer and EVP TaqMan^®^ Probe (TIBMOLBIOL, LLC, Adelphia, NJ, USA) (Table 1), was designed, using the Primer Express^®^ Software (Applied Biosystems, Foster City , CA, USA) with default parameters. To confirm the RT‐qPCR results and identify other enteroviruses and rhinovirus, DNA sequencing from amplicons was performed in the Applied Biosystems ABI PRISM^®^ (Foster City , CA, USA) 3130xl Genetic Analyzer instrument with BigDye Terminator v3·1 Cycle Sequencing kit^®^.

**Table 1:** Primers and probe used in this study

Name	Sequence	Type	Position*	Target	Protocol	Source
EV1	CAAGCACTTCTGTTTCCCCGG	Sense	167–187		Nested RT‐PCR first round	
EV2	TCCTCCGGCCCCTGAATGCG	Sense	446–465	Enterovirus sp.	Nested RT‐PCR second round and DNA sequencing	García‐Elorriaga *et al*. (2012)
EV3	ATTGTCACCATAAGCAGCCA	Antisense	600–581		Nested RT‐PCR, RT‐qPCR, and DNA sequencing	
EVD68F	GGAGCAAGTGCTCACARG	Sense	481–498	EV‐D68	RT‐qPCR	This study
EVP**	FAM‐AACCGACTACTTTGGGTGTCCGTGTTTC‐BHQ	Probe	538–565	Enterovirus sp.	RT‐qPCR	Oberste *et al*. (2010)
EVD68VP1F	GAAGCCATACAAACTCGCAC	Sense	2545–2564***	EV‐D68	Sequencing	This study
EVD68VP1R	TGGATTTATTCCATACAGACC	Antisense	3043–3062***	EV‐D68	Sequencing	This study

*According to enterovirus D reference genome NC_001430·1.

**TaqMan probe is labeled with 6‐carboxyfluorescein (FAM) and the Black Hole Quencher^®^ (BHQ^®^).

***According to enterovirus D68, strain Fermont AY426531·1.

### Partial sequencing of EV‐D68 VP1

Partial sequencing of the VP1 was performed in 15 patients, directly from respiratory samples. Briefly, 519‐bp amplicons were obtained using specific primers for VP1 (Table 1) in 25 μl reactions using the OneStep RT‐PCR Kit (QIAGEN, Valencia, CA). The amplicons were sequenced in both directions with the same primers. Sequencing reactions were performed with BigDye Terminator v3·1 (Life Technologies, Carlsbad, CA, USA) as indicated by the manufacturer. Sequences were obtained by capillary electrophoresis using an ABI Prism 3500 Genetic Analyzer (Life Technologies) and were assembled using MEGA 5·0·5. The aforementioned sequences can be found at GenBank (KR081323‐KR081333 and KT382190‐KT382193).

**Table 2:** Clinical symptoms and ventilatory support of EV‐D68 infections and its comparison with rhinovirus infection in children with acute respiratory infections in Mexico City

	EV‐D68 Infection (*N* = 24)	Rhinovirus (*N* = 16)	EV/RV Negative (*N* = 86)	*P*‐Value*
Male	45·8	62·5	60·5	0·406
Cough	91·7	100	88·4	0·339
Dyspnea	75·0	56·2	53·5	0·060
Wheezing	75·0	68·8	59·3	0·331
Rhinorrhea	54·2	31·2	65·1	0·036
Fever	50·0	50·0	66·3	0·218
Intercostal retraction	33·3	18·8	39·5	0·271
Malaise	12·5	31·2	27·9	0·262
Crackles	37·5	43·8	47·7	0·670
Conjunctival discharge	12·5	0·0	2·3	0·054
Nasal congestion	12·5	12·5	10·5	0·944
Hyporexia	8·3	6·2	19·8	0·211
Suprasternal retraction	8·3	6·2	8·1	0·964
Asthma	37·5	31·2	18·6	0·119
Pneumonia	66·7	68·8	80·2	0·292
Ventilatory support	25	20·8	10·5	0·186

*Chi‐square tests.

### Phylogenetic analysis

A total of 97 EV‐D68 VP1 partial nucleotide sequences were analyzed, including 15 sequences obtained as previously described and 82 obtained from year 1962 to 2014, available at the NCBI database. A final multiple alignment of 453 bp in length was created and manually edited with MEGA 5·05.^11^ Unrooted maximum‐likelihood tree with 1000 bootstrap replicates was constructed using the Kimura model with 5‐parameter gamma‐distributed rates. Genetic distance analysis between VP1 sequences was conducted using the Kimura 2‐parameter model^12^ in MEGA 5·0·5. Codon positions were 1st**+**2nd**+**3rd**+** non‐coding.

### Statistical analyses

Clinical and demographic data were analyzed with the statistical program SPSS version 22 (IBM Corp., Armonk, NY). For continuous‐variable comparison, Kruskal–Wallis test was used, and chi‐square or Fisher's exact test was used as appropriate to compare categorical outcomes. The probability to suffer hypoxemia (oxygen saturation (SpO_2_) <90%) was determined by Kaplan–Meier survival analyses. Differences between hypoxemia probabilities (Figure 2) were compared between the three groups by log‐rank tests. All statistical tests were two‐tailed, and a *P*‐value <0·05 was considered significant.

## Results

### Clinical and virological findings

Between September and November of 2014, a total of 126 children with respiratory disease were hospitalized at our institute. There was a 90% increase in admission for respiratory symptoms compared with the corresponding months in 2012 and 2013 based on overall admission.^13^

At least one respiratory virus was detected in 78 (62%) of NPS from the enrolled patients. The most common were enterovirus/rhinovirus (EV/RV) in 40 (31·7%) patients, followed by RSV (11·9%), HMPV (8·7%), HPIV (4·0%), adenovirus (2·4%), and influenza A and coronavirus OC43 (both with 1·6% each). Single viral infections were detected in 70 samples (55·5%), while double viral infections were found in 8 samples (6·3%).

Of the 40 patients positive for enterovirus/rhinovirus, EV‐D68 was identified in 24 patients (19%) and rhinoviruses B and C were identified in 16 patients (12·7%); EV‐D68 was identified in 12 patients in September, 10 in October, and 2 in November 2014.

Median age of patients infected with EV‐D68 was 5·2 years (IQR 1·75–7·8), and 13 (54·2%) of them were female. Common features of the hospitalized patients included cough, rhinorrhea, dyspnea, and wheezing (Table 2). Among all hospitalized patients with respiratory infection, individuals with an EV‐D68 infection had significantly greater neutrophilia (80·9%, 95% CI 65·0–92·2, *P* = 0·001) and lymphopenia (13·9% 95% CI 5·3–24·5; *P* = 0·0002) when compared with those negative for enterovirus/rhinovirus infection, but not when compared with those positive for rhinovirus infection (Table 3). Other parameters such as O_2_ saturation on room air, partial oxygen and carbon dioxide pressure, bicarbonate, oxygen saturation, and leukocyte, monocyte, eosinophil and basophil levels did not show any significant differences (Table 3). To find a relationship between EV‐D68 infection and wheezing/asthma or pneumonia diagnoses, we classified patients into two groups. Among the 24 patients positive for EV‐D68 infection, nine (37·5%) were admitted because of wheezing or asthma exacerbation with no lung opacities in the chest roentgenogram and 15 (62·5%) had opacities (pneumonia), and we found no significant difference between both groups.

**Table 3:** Laboratory findings of EV‐D68 infections and its comparison with rhinovirus infection in children with acute respiratory infections in Mexico City

Laboratory findings	Positive Enterovirus/Rhinovirus						Negative Enterovirus/Rhinovirus (*N* = 86)			*P*‐Value*
	Enterovirus D68 (*N* = 24)			Rhinovirus (*N* = 16)						
	Median	Q1	Q3	Median	Q1	Q3	Median	Q1	Q3	
O_2_ saturation on room air	87	84	91	86	82	88	87	84	89	0·321
PaCO_2_ (Torr)	27·6	25·1	33·8	30·7	28·0	34·6	31·6	27·4	34·9	0·286
PaO_2_ (Torr)	55·2	49·2	65·3	49·1	37·6	55·3	53·8	47·2	58·8	0·086
HCO_3_ (mMol/L)	17·0	15·5	20·0	19·1	16·1	20·8	17·8	16·0	20·2	0·360
SaO_2_ (%)	86·9	81·3	91·6	83·5	77·4	88·0	86·0	79·6	89·9	0·354
PaO_2_/FiO_2_ (Torr)	262	234·4	285·2	225·5	170·8	258	252·4	221·2	276·4	0·051
Leukocytes (10^3^/mm^3^)	12·1	8·9	15·0	12·1	9·8	17·6	9·7	6·9	12·9	**0·031**
Neutrophils (10^3^/mm^3^)	8·0	7·1	11·9	8·5	6·0	13·9	5·7	3·7	8·9	**0·003**
Neutrophils (%)	80·9	65·0	92·2	73·6	56·9	83·5	61·5	48·9	74·0	**0·001**
Lymphocytes (10^3^/mm^3^)	1·4	0·8	2·9	2·1	1·6	2·7	2·4	1·7	3·9	**0·026**
Lymphocytes (%)	13·9	5·3	24·5	16·3	10·7	31·9	29·2	17·6	39·7	**0·000**
Monocytes (10^3^/mm^3^)	0·4	0·1	1·5	0·9	0·6	1·1	0·8	0·4	1·1	0·315
Monocytes (%)	4·3	1·7	9·8	7·6	3·8	9·5	7·8	5·1	9·8	0·077
Eosinophils (10^3^/mm^3^)	0·00	0·00	0·1	0·08	0·00	0·25	0·00	0·00	0·1	0·197
Eosinophils (%)	0·35	0·00	1·97	0·35	0·00	1·48	0·2	0·00	0·7	0·644
Basophils (10^3^/mm^3^)	0·00	0·00	0	0·01	0·00	0·1	0·00	0·00	0·07	0·052
Basophils (%)	0·20	0·1	0·38	0·30	0·30	0·4	0·30	0·20	0·5	0·079

PaO_2_, partial oxygen pressure; PaCO_2_, carbon dioxide partial pressure; HCO_3_, bicarbonate; SaO_2_, oxygen saturation; Q, quartile.

*Kruskal–Wallis test. Significant values with *P* < 0·05 are shown in bold.

Sixteen of the 24 EV‐D68‐positive children (67%) were febrile. No neurological signs or symptoms as such acute flaccid paralysis, encephalitis, or aseptic meningitis were observed in the hospitalized children included in this study.

**Figure 1:**
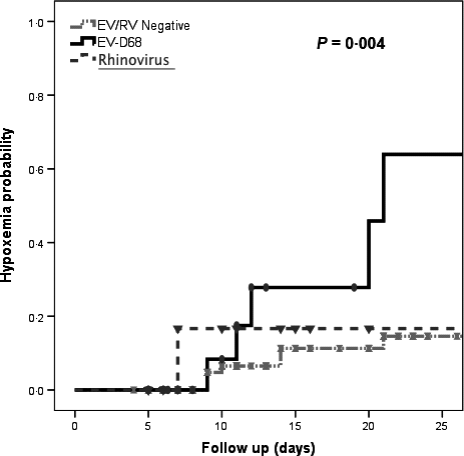
Kaplan–Meier curves of hypoxemia probability according picornavirus infection. Patients rhinovirus‐ and enterovirus D68‐negative (EV/RV–negative, *N* = 86), and patients rhinovirus‐positive (*N* = 16) and enterovirus D68‐positive (*N* = 24). Hypoxemia probability was defined in Methods.

The length of hospital stay for all the EV‐D68‐infected patients was 6 days (median), IQR (4–7), while the whole evolution period (considered from the symptom onset until disease resolution) was 11 days (median), IQR (7–21). Regarding respiratory parameters, most admitted children required significant levels of respiratory support. Patients with EV‐D68 infection were significantly more likely to progress to hypoxemia than those with rhinovirus infection or those who were enterovirus/rhinovirus‐negative (*P* = 0·044) (Figure 1).

### Molecular characterization of enterovirus D68 isolates

**Figure 2:**
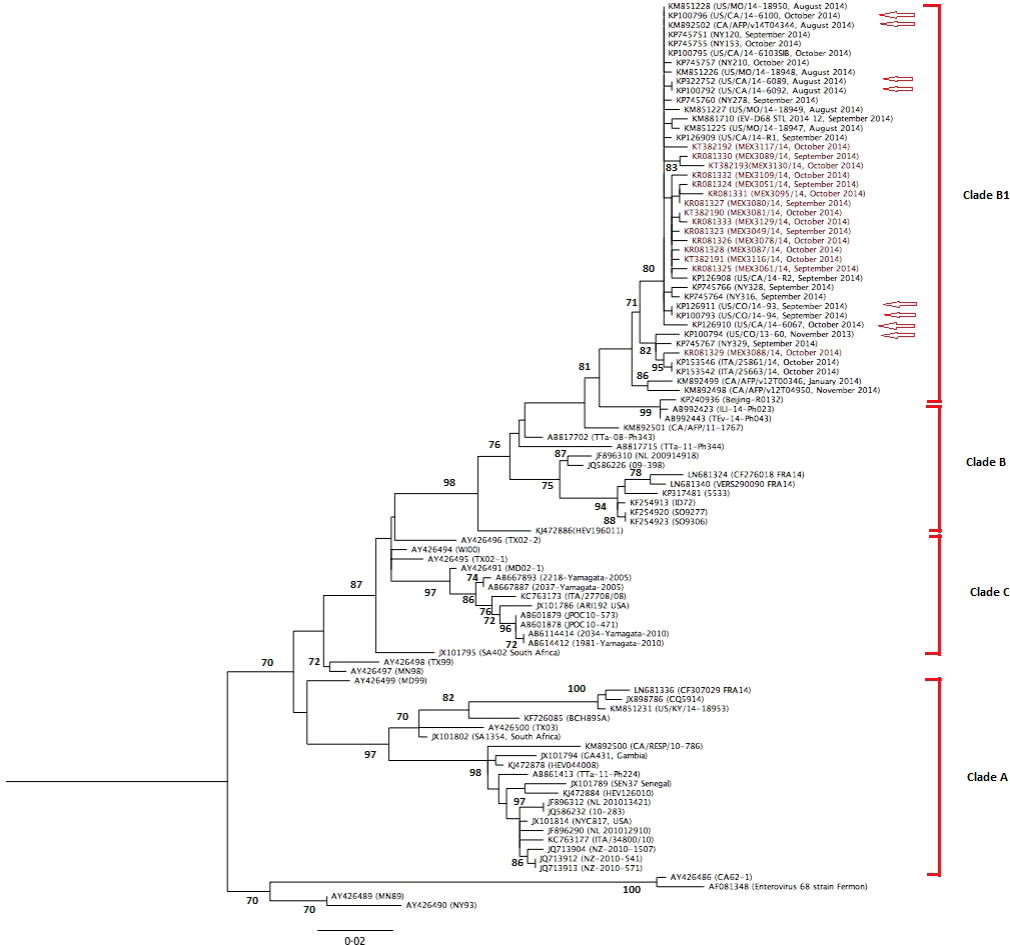
Maximum‐likelihood (ML) phylogenetic tree for EV‐D68 VP1 region. ML tree from 97 enterovirus D68 sequences registered in GenBank was calculated using partial VP1 region (453 bp) with 1000 bootstrap replicates. The sequence of 15 isolates from Mexico 2014, and eight strains from patients with acute flaccid myelitis were included (red arrows). Clades of EV‐D68 are indicated with square brackets. Bootstrap values (>70) are shown in each node.

To characterize enterovirus D68 strains associated with children with acute respiratory diseases, we obtained 15 partial VP1 sequences. Of the remaining samples, six were negative for VP1 amplification and three were positive; however, the quality and quantity of genetic material available were not enough to obtain a readable sequence. Phylogenetic analysis based on 453 nucleotides of VP1 showed that EV‐D68 prevalent in Mexico during 2014 (Figure 2) formed a robust cluster with sequences of strains isolated during 2012–2014 in New York, California, Colorado, and Missouri, USA, and also with sequences from Italy. The EV‐D68 formed 3 different clades as previously reported; however, the most recently isolates including the acute‐flaccid‐myelitis‐associated enterovirus D68 belonged to this recent cluster (clade B1). Mexican sequences also belonged to this new clade B1 (Figure 2). Evolutionary divergence analyses showed a lowest divergence (0·005–0009) between 2014 Mexican isolates and California, New York, and Missouri sequences, with the exception of MEX3088/14 isolate which showed lowest divergence with Italian isolates (0·011).

## Discussion

We reported the first confirmed geographical and temporal outbreak of enterovirus D68 in Mexico City. Previous results^13^ showed that the infection with this enterovirus serotype has remained almost undetectable at least in the last 3 years, differently from the Netherlands where EV‐D68 seems to circulate every year in a seasonal pattern.^14^ Importantly, we observed an unexpectedly high number of detections of EV/RV during the same time frame (2012–2013) as well as high number of EV‐D68‐positive cases. These data are lower, compared with 36% of EV‐D68 cases tested by CDC in 49 states from August 2014 to January 2015.^15^ This is the first time that we detect an increase in respiratory diseases related to EV‐D68, somewhat expected due to the closeness to the United States and the continuous flow of individuals between the two countries. Molecular analysis of EV‐D68 from Mexico City tended to group with US sequences, supporting this hypothesis.

EV‐D68 has been classified into three clades: A, B, and C,^16^ but recently a distinct clade B1 emerged.^17^ Strains from the United States and Europe during 2014 belong to this clade, including viruses associated with neurological illness. However, this does not mean that all B1 EV‐D68 are greatly pathogenic, as other viruses belonging to this clade cause mild respiratory illness including EV‐D68 from Mexico.

The presence of EV‐D68 contributed to the burden of respiratory infections in our institute during the autumn of 2014. However, the number of cases decreased quickly, being undetectable at early December. All patients analyzed were admitted with pneumonia or wheezing regardless of the virus found, in contrast to previous reports in which previous history of asthma or wheezing seems to be an important severity factor.^7^ EV‐D68 infection was associated with lymphopenia and higher propensity to hypoxemia, and these clinical and hematological findings have not been reported in other populations so far. Moreover, an association between EV‐D68 and neurological disease was not detected in any positive patient.

The clinical manifestations of EV‐D68 have been reported previously, based in cohorts from the Netherlands, Philippines, and Arizona, as well CDC surveillance data: The predominant symptoms were cough and dyspnea.^18^ The majority of the patients had mild diseases, but children were identified with additional symptoms like wheeze and retraction without fever or other systemic symptoms. Our pediatric patients behave similarly, and were afebrile and had more dyspnea, wheezing, and conjunctival discharge than children with rhinovirus infection or other viral infection. Children with EV‐D68 infection had abnormal blood counts with neutrophilia and in our patients a decreased lymphocyte count but not statistically different from that found in children with rhinovirus infection.

To date, CDC has reported 1153 subjects with EV‐D68 infection, of which 14 died.^15^ Cases with neurological complication have been reported in Europe and North America; thus, EV‐D68 could emerge as a cause of poliolike syndromes, in the absence of poliovirus infection.^16^, ^19^ In accordance with our surveillance,^13^ picornaviruses are becoming emerging respiratory agents with rhinovirus C and EV‐D68 causing pneumonia and asthma exacerbations mostly in pediatric patients. Studies on EV‐D68 molecular and immunological responses and antigens,^20^ principally B1 clade, are needed to determine viral factors of pathogenesis.

Finally, the methodology proposed in this study allows the molecular identification of different enteroviruses by DNA sequencing and also the specific identification of the emerging EV‐D68 in the same viral genomic region. Rapid diagnosis through RT‐qPCR may be used as a primary screening and may contribute to proper patient management.

## Conflict of interest

All authors declare that they do not have any competing interests.

## Ethical approval

The Science and Bioethics Committee of the INER revised and approved the protocol (B‐2613) and the consent procedure.
